# A Spiking Neural Network Model of Depth from Defocus for Event-based Neuromorphic Vision

**DOI:** 10.1038/s41598-019-40064-0

**Published:** 2019-03-06

**Authors:** Germain Haessig, Xavier Berthelon, Sio-Hoi Ieng, Ryad Benosman

**Affiliations:** 1 Sorbonne Universite, INSERM, CNRS, Institut de la Vision, 17 rue Moreau, 75012 Paris, France; 2 University of Pittsburgh Medical Center, Biomedical Science Tower 3, Fifth Avenue, Pittsburgh, PA USA; 3 Carnegie Mellon University, Robotics Institute, 5000 Forbes Avenue, Pittsburgh, PA 15213-3890 USA

## Abstract

Depth from defocus is an important mechanism that enables vision systems to perceive depth. While machine vision has developed several algorithms to estimate depth from the amount of defocus present at the focal plane, existing techniques are slow, energy demanding and mainly relying on numerous acquisitions and massive amounts of filtering operations on the pixels’ absolute luminance value. Recent advances in neuromorphic engineering allow an alternative to this problem, with the use of event-based silicon retinas and neural processing devices inspired by the organizing principles of the brain. In this paper, we present a low power, compact and computationally inexpensive setup to estimate depth in a 3D scene in real time at high rates that can be directly implemented with massively parallel, compact, low-latency and low-power neuromorphic engineering devices. Exploiting the high temporal resolution of the event-based silicon retina, we are able to extract depth at 100 Hz for a power budget lower than a 200 mW (10 mW for the camera, 90 mW for the liquid lens and ~100 mW for the computation). We validate the model with experimental results, highlighting features that are consistent with both computational neuroscience and recent findings in the retina physiology. We demonstrate its efficiency with a prototype of a neuromorphic hardware system and provide testable predictions on the role of spike-based representations and temporal dynamics in biological depth from defocus experiments reported in the literature.

## Introduction

The complexity of eyes’ inner structure implies that any visual stimuli from natural scenes contains a wide range of visual information, including defocus. Several studies have shown that defocus is essential in completing some tasks and more specifically for depth estimation^1,2^. Although a large body of research on Depth From Defocus (DFD) exists since the early 60’s, there is currently a gap between the information output from biological retinas and the existing literature both in the vision science and computer vision that uses images as the sole source of their studies. Although images are perfect to display static information, their use in acquiring dynamic contents of scenes is far from being optimal. The use of images implies a stroboscopic acquisition of visual information (unknown to biological systems) at a low sampling frequency. They are thus unable to describe the full dynamics of observed scenes. On the other hand, retinal outputs are massively parallel and data-driven: ganglion cells of biological retinas fire asynchronously according to the information measured in the scene^3,4^ at millisecond precision. Recent neuroscience findings show that this temporal precision can also be found in other subcortical areas, like the lateral geniculate nucleus (LGN)^5,6^ and the visual cortex^7^. The last decade has seen a paradigm shift in neural coding. It is now widely accepted that precise timing of spikes open new profound implications on the nature of neural computation^8,9^. The information encoded in the precise timing of spikes allows neurons to perform computation with a single spike per neuron^10^. Initially supported by theoretical studies^11^, this hypothesis has been later confirmed by experimental investigations^12,13^.

Here, we present a novel approach to the depth from defocus, inspired by biological retina ouput, which is compatible with ultra low latency and low power neuromorphic hardware technologies^14^. In particular, we exploit advances made in both mixed signal Analog/Digital VLSI technology and computational neuroscience which enabled us to combine a new class of retina-like artificial vision sensors with brain-inspired spiking neural processing devices to build sophisticated real-time event-based visual processing systems^15–17^. We show how precise timing of spiking retinas allows the introduction of a novel, fast and reliable biologically plausible solution to the problem of estimating depth from defocus directly from the high temporal properties of spikes.

Silicon retinas located at the core of the hereby presented system are a novel piece of hardware which do not sense scenes as a serie of frames. Conventional cameras wastefully record entire images at fixed frame rates(30–60 Hz) that are too slow to match the temporal sub-millisecond resolution of human senses. Silicon retinas are asynchronous and clock-less, every pixel is independent from its neighbors and only reacts to changes caused by movements in a scene. Data are transmitted immediately and are scene driven, resulting in a stream of events with a microsecond time precision equivalent to conventional high-speed vision sensors, with the addition of being low power and sparse^18^. This type of acquisition increases the sensor dynamic range and reduces power computation.

Spiking Neural Networks (SNNs^19^) are computational models using neural stimulation. It has been shown that such networks are able to solve constraint satisfaction problems^20,21^, depth extraction from stereovision^22,23^ or flow computation^24,25^. As they are mimicking real neurons behavior, they allow a massively parallel, low power calculation, which is highly suitable for embedded computation. The use of a SNN in this work is a natural choice to build a complete neuromorphic event-based system, from the signal acquisition to the final output of the depth information. This is advantageous because of the resulting low-power system promised by the spiking/neuromorphic technology. The developed architecture is particularly adapted on a variety of existing neuromorphic spiking chips such as the SpiNNaker^26^, TrueNorth^27^ or LOIHI^28^ neural chips. More specific neuromorphic hardware, such as the 256 neurons ROLLS chip^29^, can also be used. When combined with an event-based camera, power as low as 100 mW is proven to be sufficient to achieve a realtime optical flow computation^25^. We are showing with this work that a low-power (≤100 mW), computationally inexpensive and realtime DFD system can be similarly achieved.

Among the multitude of techniques developed by vision scientists to estimate depth, those called *depth from focus* (DFF) or *depth from defocus* (DFD) have the great advantage of requiring only a monocular camera^30^. The DFF method uses many images, and depth clues are obtained from the sharpness at each pixel. This method is computationally expensive and the amount of data to process is substantial. On the other hand, DFD estimates the variance of spatially varying blur spots based on a physical model. This technique requires less images but at the cost of a greater error in positioning. Current methods that use DFD or DFF generate depth maps for static scenes only^31^ as they are limited by the frame rate of the camera driven at maximum of 25 fps. The computer vision and engineering community have described a number of algorithms for defocus computation^32–34^. However, they typically require multiple concurrent images^35–37^, lightfield systems^38^, specific lens apertures^35,39^, correlations^40^, specific hardware^41^ or light with known patterns projected onto the environment^37^. The use of images and luminance implies high computational costs of around 17 ms to process a single frame^40^.

These approaches cannot serve as conceivable models of defocus estimation in natural visual systems, as mammalian usually operate on a complete different data format and acquisition principles. Early studies^42,43^ show that the border between blurred and sharp regions can be used to establish the depth-order of objects. For example, an out-of-focus target with a blurry textured region and a blurry border was perceived to be located proximal to the plane of focus, while an out-of-focus target with a blurry region and a sharp border was perceived to be located distant to the plane of focus. Recent findings in neuroscience show that blur perception in human is a dynamic process that allows depth assessment. In particular, the retinal defocus blur provides information regarding the relative and/or absolute distance of objects in the visual field^44^. Recently^45^, it has been demonstrated that subjects were able to detect the relative distance of two vertical edges, justifying that the retinal blur allowed the subjects to judge target distance deferentially without any other depth cues. Other studies demonstrated that motor efference and/or sensory feedback related to the blur-driven accommodative response contain sufficient information to estimate the absolute distance of visual targets^46^. In addition, information derived from image blur can be integrated by the visual system with other visual cues (e.g., retinal disparity, size, interposition, etc.), which would assist in enabling one to judge the depth order of objects over a range of distances^43,47–50^. The addition of blur information can improve the speed and accuracy in such a depth-ordering task^51^.

## Materials and Methods

### Event based cameras

**Figure 1:**
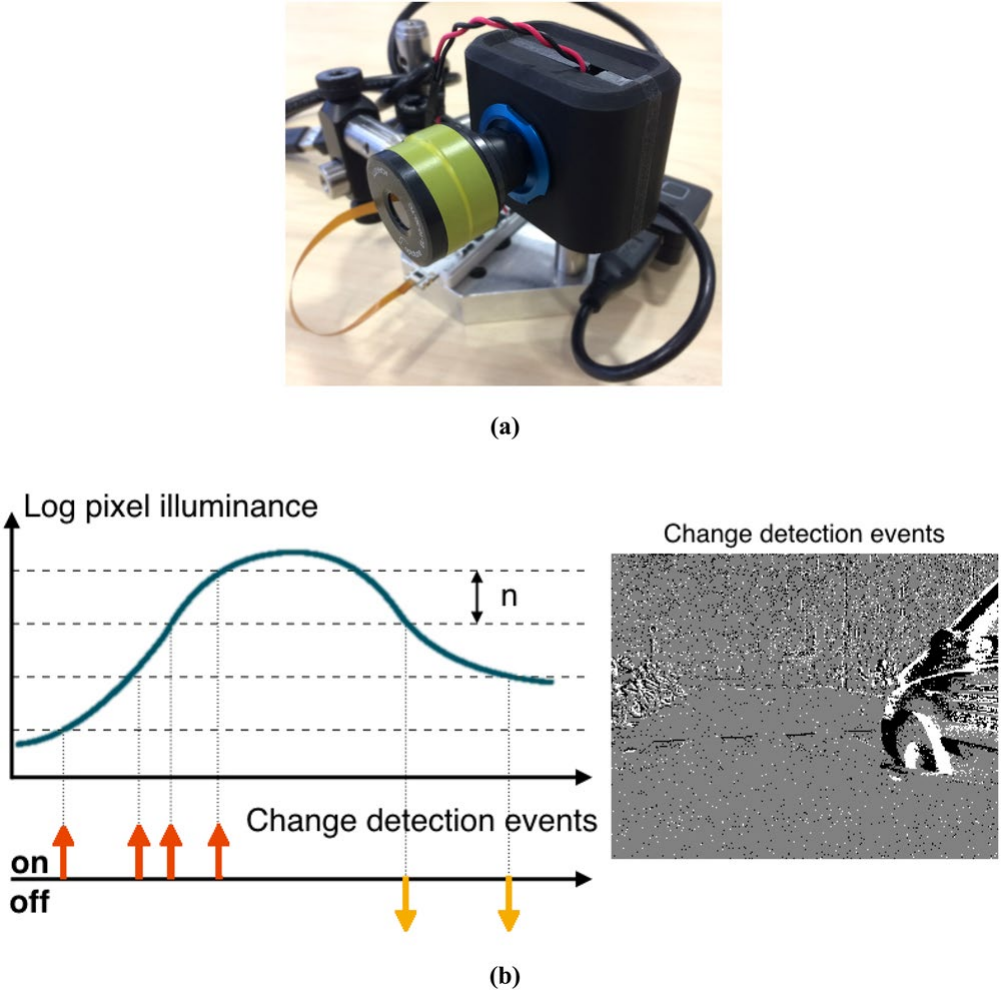
(**a**) The neuromorphic silicon event based camera with the variable motorized focal lens controlled at 100 Hz. (**b**) (left) Operating principle of event detection of an event-based camera: relative changes of the luminance greater than a predefined threshold *n* generate ON/OFF events when there is a positive/negative change of contrast. (right) Events output from the senors are shown as on the focal plane as a frame for purpose display, black dots represent OFF events while white dots represent ON events.

Biomimetic neuromorphic silicon event-based cameras are a novel type of vision sensor that are data driven. Unlike their frame-based counterparts, they are not controlled by artificially created timing and control signals (frame clock) with no relation to the source of the visual input. Events are generated when significant changes of the relative luminance occur at the pixel level as shown on Fig. 1. The visual output is in the form of an address event (AER) and encodes the visual information in the time dimension at the microsecond time precision. As soon as a change of luminance is detected, the process of communicating the event off-chip is initiated. The process executes with low latency, of the order of a microsecond, ensuring that the time at which an event is read out from the camera inherently represents the time at which a contrast change is detected. Let *e*(*x*, *y*, *p*, *t*) be an event occurring at time *t* at the spatial location (*x*, *y*)^*T*^. A positive change of contrast will result in an “ON” event (*p* = +1) and a negative change of contrast will result in an “OFF” event (*p* = −1). The threshold *n* beyond which a change of contrast is high enough to trigger an event is tuned according to the scene. Smaller intensity fluctuations do not generate any event and are not recorded. The camera used in our setup is issued from a new generation of asynchronous sensor based on^18^ and developed by Prophesee. It has a 640 × 480 pixels resolution with a high temporal resolution of 1 *μs*. This array of fully autonomous pixels combines both a luminance relative change detector circuit and a conditional exposure measurement block (not used in the paper). When no change of luminance is detected, no events are generated and the static information is not recorded. This reduces the data load and allows high speed online processing at the native resolution of the sensor.

### Depth estimation from the time of focus

**Figure 2:**
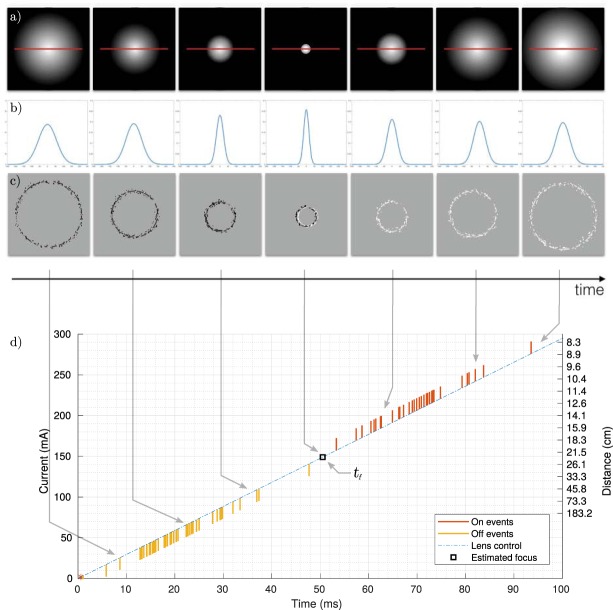
(**a**) Successive snapshots of a sphere when sweeping the focus range. The red line represents a line of pixels in the y direction. (**b**) Variations of the intensity profile along the red y-axis on the above snapshots. (**c**) Events corresponding to the sweeping of the focus range, in black are OFF events and in white ON events. (**d**) Representation of spikes among a single pixel, according to the driving current of the liquid lens. Here, the focus point is estimated to be at 22.6 cm from the sensor.

When a sweep of the focal length over its dynamic range is carried out, objects will successively appear out of focus, then in focus and out of focus again. The blurry spot around the object will therefore shrink until the object is sharp and grow again as shown in Fig. 2(a) and in Fig. 2(b) for a cross section of the blur spot. The size of the blur spot increases in connection to the distance respectively to the depth of field (DoF) location. When the object is in focus, the image spot will have its minimum size and the contrast will be maximum (sharp edges). The DoF of the lens is increasing with the distance of focus (see *Supplemental data*). Beyond a certain distance, called the hyper-focal, the whole scene appears in focus and differences in depth can no longer be distinguished. Ideally a DFD sensor should have an infinitely thin DoF for each focusing distance and an infinite hyper-focal. In practice one needs to minimize the DoF and increase the hyper-focal to have the best spatial resolution in depth on the longest distance possible.

Let *s*(*t*) be the size of the defocus blur at the focal plane. It will vary according the equation (see *Supplemental data* for details):

1 $$s(t)=\frac{{f}^{2}}{N}\times \frac{|z(t)-d|}{(d-f)z(t)},$$

with *f* the focal value of the optical system, *N* the numerical aperture, *d* the position of the object when in focus and *z*(*t*) the variable position of the object over time, or in other words the depth. Due to the aberrations, diffraction phenomenon and non-idealities of the lenses, a Gaussian point spread function (PSF) is commonly used to describe the defocus blur spot^52^. The spread parameter *σ*(*t*) is proportional to the diameter *s*(*t*) of the ideal blur circle, i.e. *σ*(*t*) = *αs*(*t*). The resulting intensity onto the sensor, at a pixel (*x*_*i*_, *y*_*i*_) is:

2 $${I}_{i,j}(x,y,t)=A.\,\exp (-\frac{{r}_{i}^{2}}{2\sigma {(t)}^{2}}).$$

with ${r}_{i}^{2}={(x-{x}_{i})}^{2}+{(y-{y}_{i})}^{2}$ and *A* the amplitude. At the pixel level the evolution of the intensity will depend on how close to the camera the object is. The Gaussian PSF is actually related to the classical formulation of the blur in the focal plane as a problem of 2D-heat diffusion. As such, the solution is the Green’s function equivalent to Eq. (2). As a function of time, the standard deviation in *I* can be used to determine the time *t* at which an event is triggered by the pixel, assuming *σ* is invertible i.e.:

3 $$t={\sigma }^{-1}(\sqrt{\frac{{r}_{i}^{2}}{2(\mathrm{log}\,A-\,\mathrm{log}\,{I}_{i,j}(x,y,t))}})$$

We are dropping subscripts (*i*, *j*) for readability purpose as what we are describing is valid for any pixel. Hence, given the intensity at an arbitrary time *t*_0_, if the variations of its log reach some threshold ±*n* (described in the previous section), then:

4 $$\mathrm{log}\,\frac{I(x,y,t)}{I(x,y,{t}_{0})}=\pm \,n\,{\rm{and}}\,\mathrm{log}\,I(x,y,t)=\,\mathrm{log}\,I(x,y,{t}_{0})\pm n.$$

This gives the time when an event is emitted according to (3):

5 $$t={\sigma }^{-1}(\sqrt{\frac{{r}^{2}}{\mathrm{2(}\mathrm{log}\,A-\,\mathrm{log}\,{I}_{0}\mp n)}})$$

The sign of *n* is chosen according to the polarity of the spiking event, itself related to the sign of the intensity’s derivative:

6 $${\rm{SIGN}}(n)={\rm{SIGN}}(p)={\rm{SIGN}}(\frac{dI}{dt})$$

when the derivative is positive the polarity will be +1 (ON event) and −1 when negative (OFF event). Eq. (5) expresses when an event will be emitted w.r.t. *n* and to a reference event measured at *t*_0_. As we reach focus, the value of *σ* will be constant for small duration of time, therefore the derivative of I, $\frac{dI}{dt}$ is equal to 0, followed by a polarity change as shown in Fig. 2(c) and expressed in the temporal domain in Fig. 2(d) around 50 ms. The detection of focus can then be determined by detecting the time *t*_*f*_ of the polarity change that can be estimated from the average timing between the consecutive ON and OFF events. We can then estimate the size of the defocus blur *s*(*t*_*f*_) according to (3) and deduce from (1), the depth information *z*(*t*_*f*_) as:

7 $$z({t}_{f})=\frac{\mp d{f}^{2}\,/\,N}{S({t}_{f})(d-f)\mp {f}^{2}\,/\,N}.$$

The change of sign in *z* corresponds to the focal length that is the closest to the focus. Parameters *d* and *f* are controls of the liquid lens device.

### Liquid lens control

The optical system shown in Fig. 1(a) is composed of three components:

- an electrically focus-tunable liquid lens with a 10 mm clear aperture and focus range *f*_*ll*_ ranging from 50 to 120 mm^53^.
- an offset lens with a focal *f*_*o*_ = −150 mm. It acts as a relay imaging system between the focus-tunable lens and the objective and ensures a proper focus.
- an objective lens with focal length *f*_*ol*_ = 35 mm, *f*_*ol*_/2 objective lens. This objective is a good compromise between large focal value, large clear aperture and low bulk (23.4 *mm* length). It is used to form an image directly on the camera pixel array.

The electrically focus-tunable lens used for this work is a shape-changing lens, consisting of an optical fluid whose deflection property changes w.r.t. the pressure applied on it via an electromagnetic actuator (coil). The focal distance is then controlled by the amount of current injected in the coil. More details can be found in Supplemental data^53^.

The thin lens approximation is given as follows:

8 $$d={f}_{eq}+\frac{{f}_{eq}^{2}}{{D}_{cam/obj}-{f}_{eq}},$$

where *d* is the position of the point in focus, *f*_*eq*_ is the global optical system’s equivalent focal value (liquid lens + offset lens + objective lens) and *D*_*cam*/*obj*_ is the distance between the camera and the object. This technique is not specific to tunable liquid lens i.e. a mechanical focus controlled lens would also work as well if we can change the focus at a high enough frequency. However mechanical device has usually a reduced operational frequency and a shorter lifetime.

### Spiking neural network

**Figure 3:**
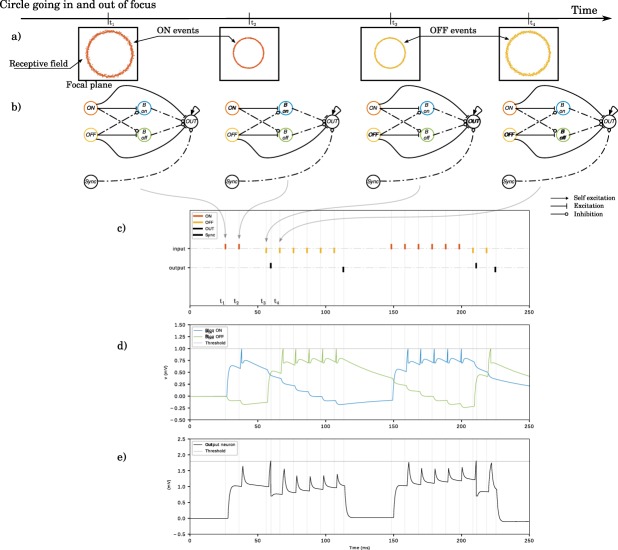
Spiking neural network. (**a**) Input data: a circle going in and out of focus, in front of a receptive field (a single pixel). (**b**) Neural network for focus detection composed of two input neurons, *ON* and *OFF*. They directly connect to the output neuron, and also to two blocker neurons *B*_*on*_ *and B*_*off*_ that are inserted to avoid parasite firings of the output neuron due to a sequence of only ON or OFF polarity events. A synchronization with the liquid lens via the *Sync* neuron is added, in order to encode the depth in the length of the spike train. (**c**–**e**) Simulation of the SNN with NEST. (**c**) The input spikes (ON and OFF events) and the output of the network (OUT and Sync). The point of focus is given by the OUT neuron, while the distance is encoded in the timing between the OUT and SYNC spikes. (**d**) Membrane potential for the two blockers neurons. After the first spike of its respective polarity, the blockers send a inhibition to the output neuron. (**e**) Membrane potential of the output neuron. Spikes from the same polarity do not allow the output neuron to reach its firing threshold, while a succession of ON and OFF events make the output neuron fire. As the output neuron is self-excitatory, the output spike train will be maintained until the strong inhibition from the synchronization comes.

To estimate *t*_*f*_ for each pixel, we are looking for the smallest time interval between two consecutive events of opposite signs. We implement a Spiking Neural Network (Fig. 3a) based on Leaky Integrate and Fire neurons^54^ to process the spikes from the output of the neuromorphic silicon retina. When the membrane potential of the neuron reaches a threshold (spiking threshold, as in Fig. 3(d) and (e)), a spike is generated and the membrane potential is reset to a rest value. For every pixel, five neurons are required. Figure 3a shows events generated by a circle going in and out of focus. At time *t*_1_, the stimulus in front of the receptive field generates a ON event (orange - Fig. 3c). The synaptic weight between the ON and *B*_*on*_ neurons is not strong enough to trigger yet the *B*_*on*_ neuron (Fig. 3d). As a second spike is generated by the same neuron at time *t*_2_, the *B*_*on*_ neuron reaches its threshold value and spikes (Fig. 3d). An inhibition link to the OUT neuron ensures that the OUT neuron won’t fire now. After the focus, at time *t*_3_, we have a polarity inversion: the OFF neuron fires, thus exciting the output neuron that fires (Fig. 3e). The next OFF spike, at time *t*_4_, activates the *B*_*off*_ neuron, thus preventing the OUT neuron to fire again in response to the future OFF spikes. Finally, the Sync neuron is triggered by the liquid lens, warning that the sweep is over and resetting the OUT neuron to its initial state. The depth can then be extracted as the timing between the OUT and Sync spikes. The neural architecture shown in Fig. 3b is sharing some similarities with the one presented in^55^ to measure contrast. However, they are both fundamentally different as one is focusing on measuring spatial contrast with no consideration to the temporal domain, while the other detects the maximum contrast in time and in space, by detecting the shortest duration between polarity changes.

## Results

Results are obtained for a field of view of 15° and a depth that ranges from 0.12 to 5.5 m. The distance upper bound corresponds to the hyper-focal distance of the global optical setup. The sparse nature of the data allows the algorithm to operate in real time at the native resolution of the sensor (640 × 480 pixels).

The Spiking Neural Network previously described in section 3 was implemented using the PyNN framework^56^ and simulated using the NEST neural simulator^57^. All neurons are modeled as Leaky Integrate-and-Fire (LIF) neurons. Results are presented on Fig. 3. We set the dimension of the network to fit a region of 447 × 447 pixels, the network then using 999045 neurons. This amount is compatible with existing neuromorphic hardware implementation on the TrueNorth platform (1 million neuron^27^) or SpiNNaker capability^26^.

**Figure 4:**
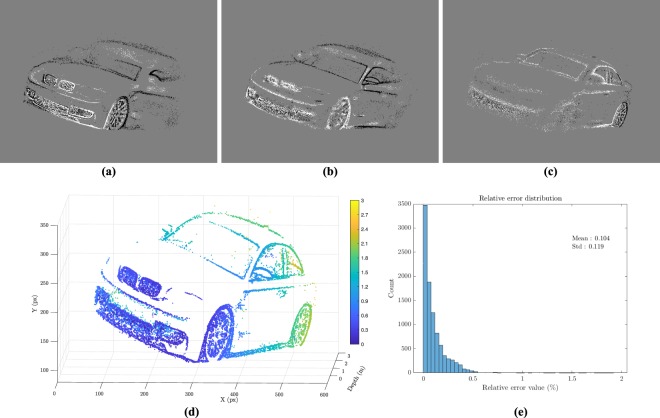
(**a**–**c**) Snapshots during a sweep of an object (**d**) Reconstructed depth scene for the car. The depth is also color-coded for clarity. (**e**) Distribution of the error. The mean relative error is at around 0.1% and a standard deviation of 0.12%.

To better understand the possibilities and limits of the system, we performed a simulation on synthetic data generated with a controlled optical setup where all parameters can be tuned. The aim of this simulation is to study the algorithm without constraints from the physical setup. Figure 4 shows three snapshots of the events generated during a sweep of a car. Figure 4d shows the reconstructed depth computed by the system.

All the parameters being known we can estimate the relative error to the ground truth. We notice that most of the error is located at the front of the car on the grating where close to one another straight lines patterns are located. This is a known limitation of several vision algorithms such as stereo matching, which will be further discussed in section 3.1. Figure 4(e) displays the error repartition with a mean relative error of 10.4%. An example video on a car is available online^58^.

**Figure 5:**
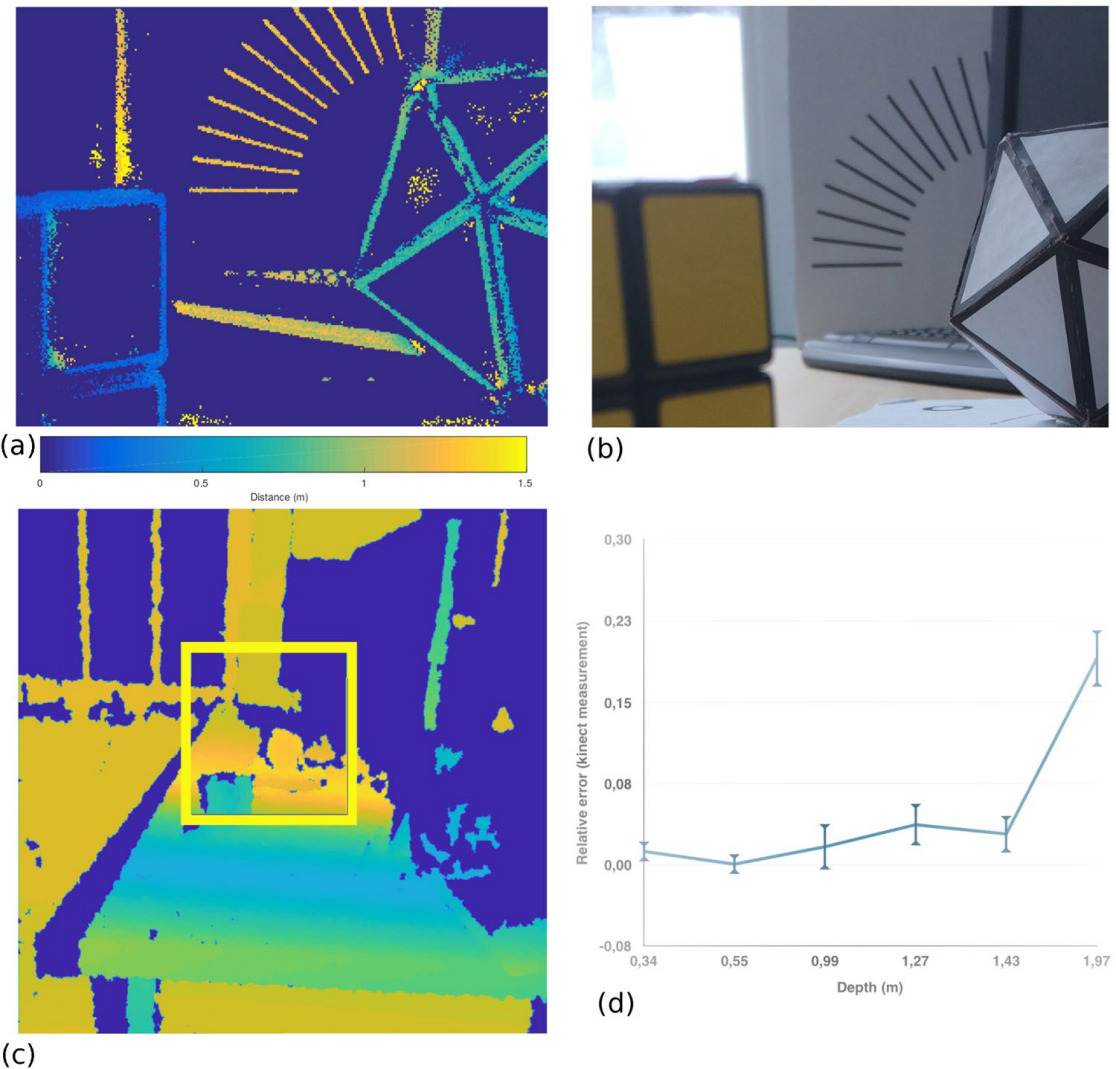
(**a**) Depth map from the developed setup (raw data, no post-processing). (**b**) Conventional Image of scene for display purposes. (**c**) Depth map from the Kinect used as reference. The yellow square corresponds to the field of view. (**d**) Relative error for this scene, with the output of a Microsoft Kinect as ground truth. A sparse set of handpicked points were selected in the ground truth and then compared to depth estimations from our network.

The second experiment, the depth estimated from the DFD is assessed with our setup on a monitored scene where the ground truth is provided by a Microsoft Kinect sensor. The Kinect is taken as the reference similarly to previous studies^59,60^, reporting reconstruction precision of few mm at 50 *cm* to 3 *cm* at 3 *m*. Figure 5 shows the setup and the depth map computed for the presented neuromorphic technique with a comparison with the groundtruth depth map: the error is increasing relative to depth. Up to 1.5 *m*, the relative error is upper-bounded at 4% and increased up to 23% at 2 *m*. This is however an expected result as the optical system’s focal length is reaching the hyper-focal.

**Figure 6:**
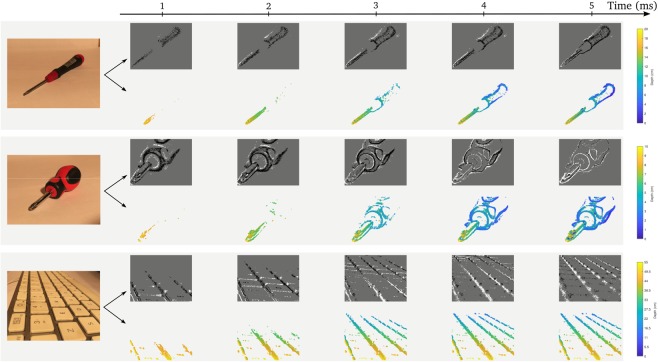
Snapshots of the event stream, and associated depth maps during a sweep (5 ms) for multiple objects. Black and white dots are the OFF and ON events from the event-based silicon retina, as described in Section 2.1. Distance is color-coded.

The third experiment shows reconstruction for several objects with different textures and sizes. Figure 6 shows for each object its corresponding depth map while the lens is sweeping through the object.

### Remarks and limitations

The algorithm is inexpensive in computational power, in the presented experiments it is able to deal with around 15 million events per second. A shaky scene viewed by the event-based camera will generate at most 2 million events per second. In the worst case, thus the current approach is 7 times faster than real time. However for most objects used it is more around 20 times faster than real time using an off-the-shelf laptop. The computational load of the proposed method is lower than any other existing method because it relies on detecting changes of polarities from a sparse output while existing techniques such as^41^ require to compute the local gradient on entire frames. This algorithm can be easily embedded on portable devices such as smartphones or autonomous vehicles as an ideal method for low power solutions to obstacle side-stepping or 3D scanners. The low-cost liquid lens used in this paper consumes ~300 *mA*. New consumer ready products using electrowetting^61^ and more advanced research prototypes^62,63^ allow a low power budget of less than 1 *mW* at the cost of losing temporal accuracy.

**Figure 7:**
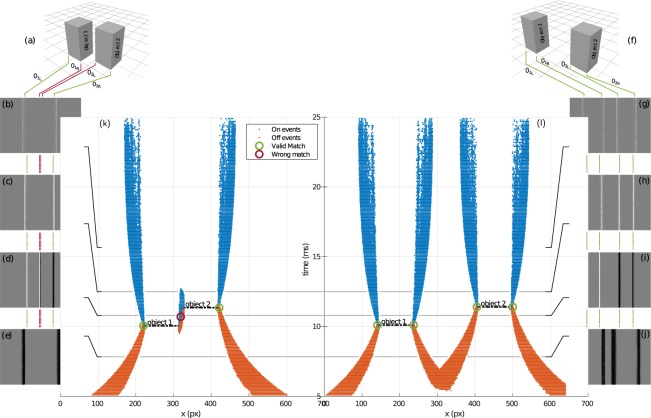
Highlighting of the wrong depth measurements for two closeby edges. The two central plots show events in the x-time plane, smashing the y-dimension. Events are color coded with their polarity (red for OFF events, blue for ON events). The right one is a valid case, with no overlap. The left one contains an overlap in the event stream, leading to wrong depth deductions in this case. 4 snapshots of events are presented for every case.

As pointed out during experiments, repetitive patterns can lead to incorrect depth estimation. Figure 7 shows this situation for simulated data. If we consider two objects that are well separated (Fig. 7f), the sweep of the liquid lens will produce an event stream (Fig. 7l) with non overlapping spikes. Figure 7j is a snapshot of the sweeps’ beginning. The four OFF edges are distinct. As the focus evolves, we reach the focus point for object 1 (Fig. 7i). The two edges *O*_1*L*_ and *O*_1*R*_ of object 1 now generate ON events. After the focus point for object 2 (Fig. 7h), the two other edges *O*_2*L*_ and *O*_2*R*_ now generate ON events. As the objects are in a sufficient relative distance, the edges *O*_1*R*_ and *O*_2*L*_ are not overlapping.

If we consider two objects that are close each other (Fig. 7a), the sweep of the liquid lens will now produce the event stream shown in Fig. 7k. As the sweep starts (Fig. 7e), only the external edges of objects 1 and 2 (*O*_1*L*_ and *O*_2*R*_) generate OFF spikes. As the focus reaches object 1, object 1 generates ON spikes and object 2 OFF spikes. The two middle edges (*O*_1*R*_ and *O*_2*L*_) are now superimposed, with two different polarities, causing the failure of the algorithm (Fig. 7d). Decreasing the size of the pixels is equivalent to increase the spatial resolution of the sensor. This will allow to estimate depth as long as we manage to separate the two edges, however the same ambiguity problem will occur once we reached the limit of the sensor. In principle as we are stimulating the same pixel a possible solution to solve this issue is to change the point of view of the camera to disambiguate depth estimation at critical locations.

## Conclusions and Discussions

In this paper we proposed a spiking neural network model that solves the depth from focus efficiently by exploiting an event-based representation amenable to neuromorphic hardware implementations. The network operates on visual data in the form of asynchronous events produced by a neuromorphic silicon retina. It processes these address-events in a data-driven manner using artificial spiking neurons computation units. This work introduces a valid explanation and a robust solution to depth estimation from defocus that has not been reported in the literature. The overall system matches recent existing literature of neuroscience, biological retinas and psychophysics studies on the role of defocus in the visual system. This network is nonetheless an abstract simplification of the depth estimation problem that must surely combine more complex information in biological systems. More importantly, this study should be coined depth from focus rather than from defocus as the neural structure developed aims at detecting the exact time of focus during a sweep.

During the last five decades of research, DFD has remained an unsolved issue. The fundamental difference and novelty of this work is that the proposed network operates using exclusively precisely-timed contrast events. These events are measured directly from the neuromorphic silicon retina, which models only the transient responses of retinal cells (i.e., of the Y-ganglion cells), without including the sustained ones, yet present in the system. While the sustained information is present in the silicon retina used, we show that this information is not necessary to provide depth estimation from defocus. Silicon retina transient responses produce single events. Their precise timing plays a crucial role in the estimation of blur and more importantly in determining when the observed object is in focus.

In contrast, the vast majority of computational models of depth from defocus are based on images that are known to be absent from the visual system and only rely on luminance information. Additionally, none of them use the precise timing of spikes. In these models, convolutions techniques are used to determine the level of blur. These methods are computationally expensive and meaningfully slower as several acquisitions are often needed to provide an accurate result. By contrast, the model we presented does not incorporate any notion of filtering or convolutions. These choices are based on the perception of spatial contrast, whereas the presented model solely responds to temporal contrast.

Whether the brain is using such a technique to estimate depth from defocus is an open question. However due to the nature of precisely timed information output by biological retinas^64^ convolutions algorithms cannot provide a viable explanation as the stroboscopic nature of image acquisition and luminance use is incompatible with neural systems. Instead, we show that the change of polarity at the pixel level contains sufficient information to estimate depth from defocus. Recent findings in physiology show that several mechanisms used by our methodology exist in Nature. Biological retinas contain several types of ganglion cells, each informing the brain about a particular content of the visual scene, such as motion, edges or chromatic composition. In a recent paper, a newly discovered ganglion cell type ‘On-delayed’ is described^65^. This cell has been shown to respond vigorously to increasing blur. Its degree of firing directly encodes the amount of high spatial frequencies contained in its receptive field. More importantly, this cell gets input from both ON and OFF polarities. While it is currently unknown how this defocus information is used by the brain, it is most likely that this information projects to the visual thalamus and cortex and also to midbrain structures where accommodation is controlled^66^.

We expect the most significant impact of our model to be in the field of artificial vision. Today’s machine vision processing systems face severe limitations imposed both by the conventional sensors front-ends (which produce very large amounts of data with fixed sampled frame-rates), and the classical Von Neumann computing architectures (which are affected by the memory bottleneck and require high power and high bandwidths to process continuous streams of images). The emerging field of neuromorphic engineering has produced efficient event-based sensors, that produce low-bandwidth data in continuous time, and powerful parallel computing architectures, that have co-localized memory and computation and can carry out low-latency event-based processing. This technology promises to solve many of the problems associated with conventional computer vision systems. However, the progress so far has been chiefly technological, whereas related development of event-based models and signal processing algorithms has been comparatively lacking (with a few notable exceptions). This work elaborates on an innovative model that can fully exploit the features of event-based visual sensors. In addition, the model can be directly mapped onto existing neuromorphic processing architectures. Results show that the full potential is leveraged when single neurons from the neural network are individually emulated in parallel. In order to emulate the full-scale network, however, efficient neuromorphic hardware device capable of emulating large-scale neural networks are required. The developed architecture requires few neurons per pixel and is implementable on a variety of existing neuromorphic spiking chips such as the SpiNNaker^26^, TrueNorth^27^ or LOIHI^28^ neural chips.

## Supplementary information

Supplemental data
