# Supplementary material for: A Spiking Neural Network Model of Depth from Defocus for Event-based Neuromorphic Vision
Source: Sci Rep. 2019 Mar 6;9:3744. doi: 10.1038/s41598-019-40064-0 (PMC6403400; doi:10.1038/s41598-019-40064-0)
Supplement: Supplementary file 1 — Supplemental data [file 41598_2019_40064_MOESM1_ESM.pdf]

A Spiking Neural Network Model of Depth from Defocus for Event-based Neuromorphic Vision

Germain Haessig[1,*,+], Xavier Berthelon[1,+], Sio-Hoi Ieng[1], and Ryad Benosman[1,2,3]

[1]Sorbonne Universite, INSERM, CNRS, Institut de la Vision, 17 rue Moreau, 75012 Paris, France.
[2]University of Pittsburgh Medical Center, Biomedical Science Tower 3, Fifth Avenue, Pittsburgh, PA.
[3]Carnegie Mellon University , Robotics Institute, 5000 Forbes Avenue Pittsburgh PA 15213-3890.
[*]benosman@pitt.edu
[+]these authors contributed equally to this work

1 Supplemental data

1.1 Basics of geometrical optics

Let us consider a single thin convex lens of focal distance f with an infinite circular aperture. As shown on the Figure 1, the rays issued from a point $z = d$ converge to form an image behind the lens onto the detector according to the geometrical optic formula:

$$m \times m' = f^2 \tag{1}$$

In a stigmatic optical system, an object point is in focus when the image formed is a point as well. Any object out of focus forms a blurry spot on the detector. We can compute the distance of the camera to the objective as a function of the focal length of our optical system and the position of the object in focus:

$$D_{cam/obj} = f + m' = f + \frac{f^2}{m} = f + \frac{f^2}{d - f}. \tag{2}$$

As this distance is fixed in our setup, increasing the focal value f results in an increase of the distance d at which an object is in focus and vice-versa. By tuning the focal value of the optical system, every plane of the 3D scene is successively in focus.

1.2 Equivalent focal length

The thin lens approximation assumes that the equivalent focal length f_{eq} of our optical system is :

$$\frac{1}{f_{eq}} = \frac{1}{f_{ll}} + \frac{1}{f_{ol}} + \frac{1}{f_o} \tag{3}$$

with f_{ol} and f_o being respectively the objective lenses and the offset focal lengths.

1.3 Defocus principle

When an object is located before or after the focus point, it will form a blurry image of size s. Geometrical optic relations (Figure 1) give :

$$s = \frac{f^2}{N} \times \frac{|x - d|}{(d - f)z}, \tag{4}$$

with N the numerical aperture of the system, f the focal length, d the position of the point in focus and z the position of the object. N is defined as the ratio of the focal length divided by the aperture of the system.

A real optical system has a finite aperture size, limited by the dimensions of the optics. The spatial resolution is also limited by the pixel size below which it is not possible to distinguish focus. This represents the circle of confusion, C, of the camera as shown on Figure 1. As a consequence, a range of several points will form an image ”in focus” (the image spot size is smaller

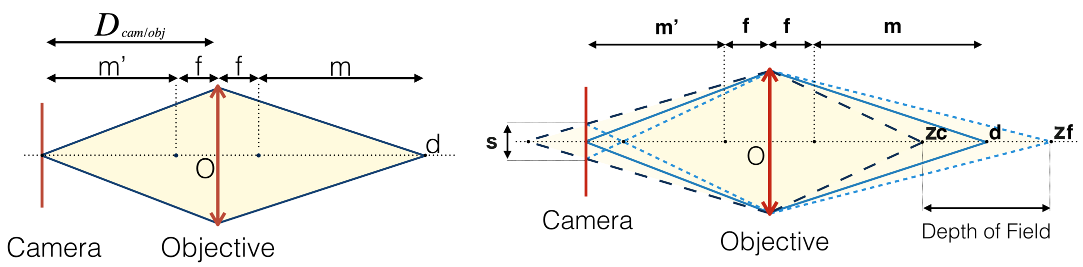

Figure 1. (left) Stigmatic optical system. (right) Principle of depth from defocus.

than the circle of confusion) on the detector for a given focal length. This range is called depth of field and the two limiting points are the values of z_{close} and z_{far} for which $s = C$, i.e.

$$z_{close/far} = \frac{d}{1 \pm \frac{CN(d-f)}{f^2}}, \tag{5}$$

The depth of field is then given by computing the difference between z_{close} and z_{far}:

$$D_oF = |zf - zc| = 2 \times \frac{CNd(d-f)}{f^2 - \frac{(CN(d-f))^2}{f^2}}. \tag{6}$$
